# Navigating the clinical landscape of artery of Percheron infarction: A systematic review

**DOI:** 10.1016/j.ensci.2024.100521

**Published:** 2024-08-21

**Authors:** Oday Atallah, Yasser F. Almealawy, Arwa Salam Alabide, Minaam Farooq, Vivek Sanker, Suraa N. Alrubaye, Rami Darwazeh, Wireko Andrew Awuah, Toufik Abdul-Rahman, Ahmed Muthana, Aalaa Saleh, Jack Wellington, Amr Badary

**Affiliations:** a Departemnt of Neurosurgery, Hannover Medical School, Hannover, Germany; b Faculty of medicine, University of Kufa, Kufa, Iraq; c Facility of medicine, Al_Kufa university, Najaf, Iraq; d Department of Neurosurgery, King Edward Medical University, Mayo Hospital, Lahore,Pakistan; e Department of Neurosurgery, Trivandrum Medical College, Kerala, India; f College of Medicine, University of Babylon, Hilla, Iraq; g Neurosurgery department, Prime Hospital, Dubai, United Arab Emirates; h University of Babylon, Hilla, Iraq; i Faculty of Medicine, Sumy State University, Sumy, Ukraine; j College of Medicine, University of Baghdad, Baghdad, Iraq; k Faculty of Medical Sciences, Lebanese University, Beirut, Lebanon; l Department of Neurosurgery, Branford TeachingHospital NHS Foundation Trust, Bradford, UK; m Departemnt of Neurosurgery, Klinikum Dessau, Dessau-Roßlau, Germany

**Keywords:** Artery of Percheron, Infarction, Stroke, Midbrain, Thalamus

## Abstract

**Introduction:**

Infarction of the artery of Percheron (AOP) is a rare vascular condition where a single arterial branch supplies blood to the thalamic and midbrain regions, leading to neurological deficits. The challenge lies in its often-delayed diagnosis due to its rarity and diverse clinical presentations, necessitating heightened awareness among clinicians for expedited diagnosis and appropriate therapeutic interventions.

**Materials and methods:**

All relevant studies involving patients diagnosed with infarction of AOP were retrieved from PubMed, Google Scholar, Web of Science, and Scopus. Only human studies that were published in full English-language reports were included. Included in the search were the terms “Artery of Percheron,” “infarction,” “stroke,” and “demarcation”. Age, gender, presenting symptoms, treatment, recovery time, and outcome of patients with AOP infarction were all recorded.

**Results:**

A systematic review was conducted on a total of 530 articles, out of which 130 articles met the specified requirements. The average age is 59, with men comprising 57.7% of the population. The symptoms reported were visual disturbance in 43.9% of cases and changed mental state in 77.2% of cases. Treatment options include conservative management (85.4%), thrombolysis (11.3%), and other approaches. The optimal age range for recovery is between 41 and 50 years old.

**Conclusion:**

Our study on acute AOP infarction highlights male predominance, common comorbidities like hypertension and diabetes, and prevalent symptoms including visual disturbance and altered mental state. Early recognition is crucial, with thrombolytic therapy within the critical time window showing promising outcomes. These findings offer insights for enhanced clinical management of AOP infarction.

## Highlights

• AOP infarction is an uncommon cerebrovascular accident, accounting for 0.1% of ischemic strokes and 4% of thalamic strokes.
• Altered consciousness, cognitive decline, and supranuclear vertical gaze palsies are common symptoms of AOP infarction.
• Early detection is crucial for managing severe deficiencies due to associations between several causes.

## 1 Introduction

Considering the neurophysiological significance of the thalamus, a rare form of stroke may jeopardize its cerebrovascular blood supply. There exist variations in said vasculature to the brainstem and thalamus. Merely 4–12% of the population has the artery of Percheron (AOP), making it one of the uncommon variations. Gérard Percheron, a French neurologist, originally reported it in 1973. He stated that it originated from the proximal posterior cerebral artery (PCA), which supplies the paramedian thalamus and rostral midbrain with bilateral artery [1].

Bilateral thalamic infarcts precipitated by AOP infarction may or may not involve the midbrain. Research indicates that this accounts for 0.1% of ischemic and 4% of thalamic strokes, respectively, suggesting such a rarity of this cerebrovascular accident [2,3]. As the thalamus is involved in a plethora of neurophysiological functions, it may be challenging to diagnose the presentation. Nonetheless, altered consciousness, cognitive decline, and supranuclear vertical gaze palsies are the most common symptoms of AOP infarction [4,5].

Depending on the pathophysiological etiology, the management of AOP infarction varies. Although dependent on the severity of the infarction, the prognosis is generally favorable in terms of mortality and long-term neurological sequalae [6]. Thus, the aim of this systematic review is to provide a comprehensive overview of the diagnosis, clinical symptomatology, and management options available for AOP infarction. Additionally, our study represents the most comprehensive review to-date of AOP infarction, which was not discussed systematically before.

## 2 Methods

Moreover, the data extracted from selected publications underwent a rigorous evaluation encompassing multifaceted variables such as age, gender distribution, employed treatment modalities, and other significant factors shaping the complex landscape of AOP. This comprehensive approach facilitated a nuanced understanding of the intricacies surrounding AOP across various dimensions.

Our systematic review of AOP commenced with a meticulous examination of diverse facets, encompassing patient demographics, etiological variables, clinical presentations, diagnostic methodologies, treatment approaches, and subsequent outcomes. Adhering rigorously to the PRISMA guidelines, our methodology ensured a comprehensive analysis.

### 2.1 Search strategy

To ensure inclusivity, an extensive search spanned four key online databases—PubMed/MEDLINE, Google Scholar, Web of Science, and Scopus—without imposing any timeframe restrictions. A refined set of keywords and Mesh phrases: “Artery of Percheron,” “infarction,” “stroke,” and “demarcation,” was tailored to optimize search precision and breadth.

In alignment with our research focus, the review specifically targeted human studies published in the medium of the English language and granting access to full-text content. Articles that did not meet the above-mentioned criteria were excluded.

### 2.2 Screening of studies and data extraction

**Fig. 1:**
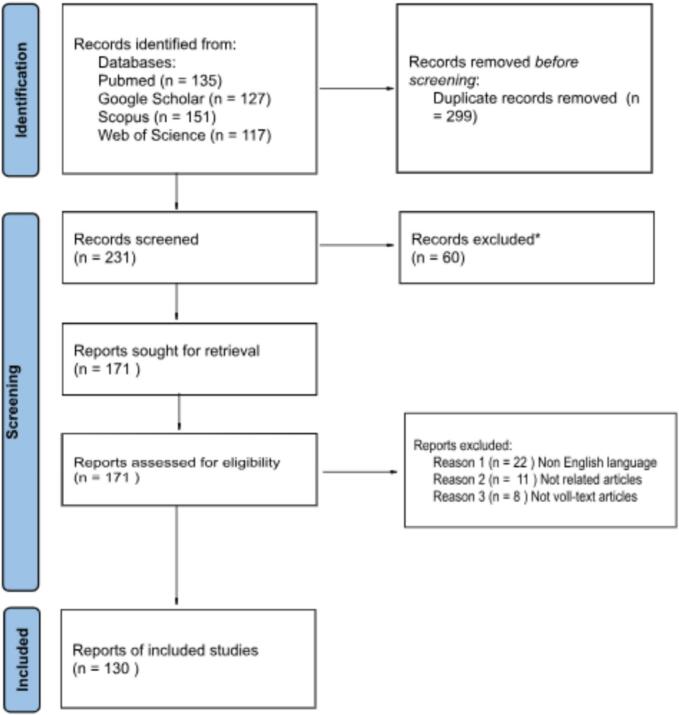
PRISMA Flow-diagram of the related articles.

Our screening process commenced with an initial assessment of study titles and abstracts to gauge alignment with our research scope. Subsequently, after eliminating duplicate entries, Two independent authors (O.A. and Y.A.) extracted relevant data from selected studies. The data collected included information such as study design, participant demographics, and the number of participants with respective outcomes and complications. Discrepancies in data extraction were resolved through consensus, and any unresolved disagreements were addressed by involving a third reviewer (O.B.) (Fig. 1).

### 2.3 Data analysis

The data was subjected to analysis, encompassing several variables such as age, gender, presenting symptoms, imaging findings, strategies for treatment, clinical outcomes, and subsequent follow-up. The analysis was done using the SPSS 26. Raw data extracted from studies was used as numeric inputs and accordingly scaled, nominated or ordinated. The frequencies, mean, median and the quartiles were analyzed. The significance of the results was delivered by using the Chi-Square in the cross tables.

### 2.4 Quality assessment

The quality assessment was performed using the JBI Checklist for Case Reports. Each article was screened twice and no article was excluded following the assessment.

## 3 Results

Initially, 530 articles were drawn from four databases. After a thorough review of titles, abstracts, and full texts, 130 papers met the inclusion criteria. This study investigated patient-level data from diverse research designs involving 279 individuals diagnosed with AOP infarction (Table 1).

**Table 1:** Supplementary table of the papers that were included.

Nr.	Author (Last name)	Gender	Age	Comorbidities/Risc factors		Duration of symptoms (days)	Symptoms			Neurological status		
				Arterial hypertension	Diabetes mellitus		Visual Symptoms - Gaze Palsy	Altered Consciousness	Memory Deficits	Consciousness	Paresis	Anisocoria
1	Agrawal 2019 [40]	F	68	Yes	Yes	<1	Yes	Yes	No	Unconscious	Quadriparesis	Yes
2	Sharma 2021 [41]	M	79	No	No	2	No	No	No	Conscious	Left hemiparesis	No
3	Almamun 2015 [4]	M	70	Yes	No	<1	No	Yes	No	Unconscious	Quadriparesis	Yes
4	Matsumoto 2022 [42]	M	74	No	No	NA	Yes	Yes	No	Unconscious	Quadriparesis	No
5	Coelh 2018 [43]	M	56	Yes	No	5	No	No	Yes	Conscious	Left hemiparesis	No
6	Baloyannis 2009 [44]	M	38	No	No	<1	No	Yes	Yes	Unconscious	No	No
7	Xiao 2018 [20]	F	48	No	No	<1	No	Yes	No	Conscious	Quadriparesis	No
8	Adcock 2019 [45]	F	69	No	No	<1	No	No	No	Conscious	No	No
9	Tryambake 2018 [46]	F	78	Yes	No	>1	Yes	Yes	Yes	Unconscious	No	No
10	Ali 2018 [47]	M	39	No	No	<1	No	Yes	No	Unconscious	No	No
11	Ravi 2020 [48]	F	68	Yes	No	<1	No	Yes	No	Unconscious	Yes	No
12	Biswas 2022 [49]	M	37	No	No	>1	Yes	No	No	Conscious	No	No
13	Pires 2021 [50]	F	77	Yes	No	<1	Yes	Yes	No	Unconscious	No	No
14	Al Ghadeeb 2022 [51]	F	33	Yes	Yes	<1	No	Yes	No	Unconscious	No	No
15	Nasir 2023 [52]	M	68	Yes	No	<1	No	Yes	No	Unconscious	Yes	No
16	Espinosa 2018 [53]	M	59	No	Yes	<1	Yes	Yes	No	Unconscious	Yes	Yes
17	Sudhir 2014 [54]	M	48	No	No	>1	No	No	No	Conscious	No	No
18	Bin Naeem 2017 [55]	F	66	Yes	No	<1	Yes	No	No	Conscious	No	No
19	Lloyd 2021 [56]	F	54	No	No	NA	Yes	Yes	No	Unconscious	No	Yes
20	Kheiralla 2021 [57]	M	55	No	No	NA	Yes	Yes	No	Unconscious	No	Yes
21	Hegde 2016 [58]	M	40	No	No	<1	No	No	No	Conscious	Yes	No
22	Jayam-Trouth A 2015 [59]	M	64	Yes	No	<1	Yes	Yes	No	Unconscious	Yes	No
23	Raicevic R 2011 [60]	M	39	No	No	No	No	No	No	Disturbed	No	No
24	Sonawale 2017 [61]	F	58	No	No	>1	No	No	No	Unconscious	No	No
25	Ahizoune A 2023 [62]	M	37	No	No	NA	Yes	No	No	Conscious	No	No
26	M Seneviratne 2020 [63]	M	51	No	No	NA	Yes	No	No	Conscious	No	No
27	Smithason 2018 [64]	M	68	Yes	No	<1	No	Yes	No	Unconscious	No	No
28	Deng 2020 [65]	F	40	No	No	<1	No	Yes	No	Unconscious	No	Yes
29	Lapsia 2019 [66]	M	59	No	No	<1	No	No	No	Conscious	No	Yes
30	Arpita 2016 [67]	F	37	No	No	<2	Yes	Yes	No	Conscious	No	No
31	Liu 2013 [68]	M	77	Yes	Yes	<1	No	Yes	No	Unconscious	No	No
32	Chang 2012 [69]	F	85	Yes	Yes	<1	No	Yes	No	NA	No	No
33	Chan 2012 [70]	M	62	No	No	>1	No	Yes	No	NA	NA	NA
34	Ahmetgjekaj 2021 [1]	M	67	Yes	Yes	NA	Yes	No	No	Conscious	No	Yes
35	Vemulapalli 2022 [71]	F	56	Yes	No	NA	No	No	No	Conscious	No	No
36	Neuwirth 2017 [72]	M	56	No	No	NA	No	Yes	No	Unconscious	No	Yes
37	Alharbi 2023 [73]	F	58	Yes	No	<1	Yes	No	No	Conscious	Right hemiparesis	No
38	Mikesell 2018 [6]	M	72	Yes	Yes	NA	Yes	Yes	No	Unconscious	No	No
39	Vipparala 2019 [5]	F	58	Yes	Yes	<1	No	Yes	No	Unconscious	No	Yes
40	Popovic 2015 [74]	F	58	Yes	No	<1	Yes	Yes	No	Unconscious	No	No
41	DiFrancesco 2017 [75]	F	71	Yes	No	<1	No	Yes	No	NA	NA	NA
42	Banza 2021 [37]	M	88	Yes	No	<1	No	Yes	Yes	Conscious	No	No
43	Cappellari 2021 [76]	M	70	Yes	Yes	NA	No	Yes	No	Unconscious	No	No
44	Kumar 2022 [77]	M	57	No	No	<1	No	No	No	Conscious	No	No
45	Michieletti 2019 [78]	M	52	No	No	NA	Yes	Yes	No	Unconscious	No	No
46	Small 2018 [79]	F	86	Yes	No	<1	No	Yes	No	Unconscious	Yes	No
47	Cai 2017 [16]	14 m 4 F	63,22	14 Yes	5 Yes	NA	15 Yes	17 Yes	No	9	No	No
48	Boghi 2021 [80]	F	19	No	No	NA	No	No	No	Conscious	No	No
49	Sijapati 2017 [81]	F	64	Yes	No	<1	No	Yes	No	Unconscious	Left hemiplagia	Yes
50	Liu 2017 [82]	M	75	Yes	Yes	<1	No	Yes	No	Unconscious	No	No
51	Parajuli 2023 [83]	F	60	Yes	No	<1	No	Yes	No	Unconscious	Left hemiplagia	Yes
52	Sonu 2023 [84]	M	90	No	No	>1	No	No	No	Conscious	No	No
53	Danjuma 2021 [85]	M	58	Yes	No	<1	No	No	No	Conscious	No	No
54	Gutierrez-Manjarrez 2018 [19]	F	62	Yes	No	<1	No	Yes	No	Unconscious	No	No
55	Munshi 2021 [86]	M	90	Yes	No	<1	No	Yes	No	Conscious	Right hemiparesis	No
56	Shaaban 2016 [87]	M	37	No	No	<1	No	Yes	No	Unconscious	No	Yes
57	López-Martínez 2009 [88]	M	27	No	No	<1	No	Yes	No	Unconscious	No	Yes
58	Bosschaert 2010 [89]	M	40	No	No	<1	No	Yes	No	Unconscious	No	No
59	Elsharkawy 2019 [90]	M	39	No	No	<1	No	Yes	No	Unconscious	No	No
60	Jagroo 2022 [91]	M	59	Yes	No	<1	No	No	Yes	Conscious	No	No
61	Yang 2022 [92]	M	58	No	No	<1	No	No	No	Unconscious	No	No
62	Zhu 2021 [93]	M	57	Yes	No	>1	No	No	Yes	Unconscious	No	Yes
63	Ochoa 2014 [3]	8 M 7F	48	5 Yes	2 Yes	NA	9 Yes	9 Yes	2 Yes	9 Unsonscios	10 Motor deficit	No
64	Moretti 2017 [94]	F	57	No	No	<1	Yes	Yes	No	Unconscious	No	No
65	Boyle 2017 [95]	M	77	No	No	NA	No	Yes	No	Unconscious	No	No
66	Chang 2017 [96]	F	36	No	No	Yes	Yes	Yes	No	Unconscious	Quadriparesis	Yes
67	Henninger 2011 [97]	M	52	No	No	NA	Yes	No	No	Unconscious	No	No
68	Tremolizzo 2020 [98]	M	60	No	No	<1	No	Yes	No	Unconscious	No	Yes
69	Agildere 2013 [99]	F	82	Yes	Yes	<1	No	Yes	No	Unconscious	No	No
70	Sengupta 2017 [100]	M	81	No	No	<1	No	Yes	No	Unconscious	No	Yes
71	Shrestha 2021 [101]	M	68	NA	NA	NA	Yes	No	No	Unconscious	No	No
72	Kesserwani 2021 [102]	F	81	Yes	No	>1	No	No	No	Conscious	No	No
73	Hruby 2008 [103]	M	62	No	No	NA	No	Yes	No	Unconscious	No	No
74	Kuar 2016 [104]	M	60	No	No	<1	Yes	No	No	Conscious	No	No
75	Boret 2010 [28]	M	64	No	Yes	<1	No	Yes	No	Unconscious	No	Yes
76	Caprara 2021 [105]	F	70	No	No	<1	No	No	No	Conscious	No	No
77	Zhenzhong Li 2023 [106]	M	51	No	No	<1	No	Yes	No	Unconscious	No	Yes
78	Sienkiewicz-Jarosz 2016 [107]	M	61	Yes	No	NA	No	Yes	No	Unconscious	Left hemiparesis	Yes
79	O'Brien 2012 [108]	M	46	No	No	NA	No	Yes	No	Unconscious	No	No
80	Pathirage 2019 [109]	M	35	No	No	<1	No	Yes	No	Unconscious	No	Yes
81	Devi 2021 [110]	F	55	No	No	<1	No	No	No	Conscious	No	No
82	Wischmeyer 2016 [111]	M	69	Yes	No	<1	No	Yes	No	Unconscious	No	Yes
83	Ndiaye 2023 [112]	F	50	Yes	No	NA	No	Yes	No	Unconscious	Right hemiplagia	Yes
84	Almeida 2023 [113]	F	58	No	No	<1	No	No	No	Conscious	Right hemiparesis	No
85	Onder 2020 [114]	F	58	No	No	<1	No	Yes	No	Unconscious	No	No
86	Raphaeli 2006 [115]	M	56	Yes	Yes	<1	No	Yes	No	Unconscious	No	No
87	González 2014 [116]	F	49	No	No	<1	No	Yes	No	Unconscious	No	No
88	lee Js 2019 [117]	F	58	No	No	NA	No	Yes	No	Unconscious	No	No
89	Salvatierra 2011 [118]	M	58	Yes	Yes	NA	No	No	No	Conscious	No	No
90	Alegría-Loyola 2018 [119]	M	84	Yes	No	NA	No	Yes	No	Unconscious	Quadriparesis	No
91	Lee 2013 [120]	F	31	No	No	>1	Yes	No	No	Conscious	No	No
92	Budinčević 2023 [121]	F	62	No	No	<1	Yes	No	No	Conscious	Left hemiparesis	No
93	Ghirlanda 2002 [122]	F	83	Yes	No	NA	Yes	Yes	No	Conscious	No	No
94	Law-Ye 2023 [123]	M	55	NA	NA	<1	No	Yes	No	Unconscious	No	No
95	Sharma 2019 [124]	M	50	No	No	<1	Yes	Yes	No	Conscious	No	No
96	Uceda A 2019 [125]	F	69	Yes	Yes	<1	No	Yes	No	Unconscious	No	No
97	Nowacki 2017 [126]	F	57	No	No	<1	No	Yes	Yes	Conscious	No	No
98	Hsu 2011 [127]	M	30	No	No	<1	Yes	No	No	Conscious	No	No
99	Tayfun 2016 [29]	F	73	No	Yes	<1	No	Yes	No	Conscious	No	No
100	Hu 2019 [128]	F	65	No	No	<1	No	Yes	No	Unconscious	No	No
101	Desai 2023 [129]	M	33	No	No	<1	No	Yes	No	Unconscious	No	No
102	Hedger 2021 [130]	F	79	Yes	No	<1	Yes	Yes	No	Yes	No	No
103	Dere 2021 [131]	6 M 4 F	63,6	8 Yes	4 Yes	NA	6 Yes	10 Yes	No	4 Unconscious	7 Motor deficit	No
104	Turla 2019 [132]	F	76	Yes	No	<1	No	Yes	No	Unconscious	No	Yes
105	Thiruvarutchelvan 2019 [133]	F	61	Yes	No	<1	Yes	Yes	No	Conscious	No	No
106	Morsli 2023 [134]	M	51	Yes	Yes	<1	Yes	Yes	No	Unconscious	Right hemiplagia	Yes
107	Batra 2022 [135]	M	69	Yes	No	<1	Yes	Yes	No	Unconscious	No	Yes
108	Chin 2023 [136]	M	62	No	No	>1	No	Yes	No	Unconscious	No	No
109	Liza-Sharmini 2018 [137]	F	24	No	No	>1	Yes	No	No	Conscious	No	No
110	Pyun 2017 [138]	F	59	Yes	Yes	>1	Yes	Yes	No	Conscious	No	No
111	Durmaz 2019 [139]	F	70	No	No	<1	No	Yes	No	Unconscious	Right hemiplagia	No
112	Pathirana KD 2017 [140]	F	71	No	No	<1	No	Yes	Yes	Unconscious	No	No
113	Hernandez-Vara 2017 [141]	F	68	Yes	No	<1	No	No	Tremor	Unconscious	No	No
114	Wright 2021 [142]	M	62	Yes	No	<1	Yes	No	No	Coscious	No	Yes
115	Chiang 2021 [143]	F	45	No	No	<1	Yes	No	No	Conscious	Right hemiparesis	No
116	Chang 2009 [144]	M	1 month	No	No	<1	No	No	No	Conscious	No	No
117	Wang 2022 [145]	F	65	Yes	Yes	<1	Yes	Yes	No	Conscious	No	No
		F	71	Yes	Yes	<1	Yes	Yes	No	Unconscious	Yes	No
		M	68	Yes	Yes	<1	No	Yes	No	Unconscious	Yes	No
		M	66	Yes	Yes	<1	No	Yes	No	Unconscious	Yes	No
		M	63	Yes	No	<1	No	Yes	No	Unconscious	Yes	No
		M	69	Yes	Yes	<1	No	Yes	No	Conscious	Yes	No
		M	45	Yes	Yes	<1	No	Yes	No	Conscious	No	No
		M	64	Yes	Yes	<1	No	Yes	No	Conscious	Yes	No
		M	60	Yes	Yes	<1	No	Yes	No	Conscious	Yes	No
		M	67	Yes	Yes	<1	Yes	Yes	No	Unconscious	Yes	No
		F	29	No	No	<1	No	Yes	No	Unconscious	No	No
		F	43	No	No	<1	No	Yes	No	Unconscious	Hemiplagia	No
		M	77	Yes	Yes	<1	Yes	Yes	No	Unconscious	Yes	No
		F	55	Yes	Yes	<1	No	Yes	No	Conscious	Yes	No
		M	60	Yes	No	<1	Yes	Yes	No	Conscious	No	No
		M	67	Yes	Yes	<1	No	Yes	No	Conscious	No	No
		M	53	No	No	<1	No	Yes	No	Conscious	Yes	No
		F	64	Yes	Yes	<1	Yes	Yes	No	Conscious	Yes	No
		M	66	Yes	No	<1	No	Yes	Yes	Conscious	No	No
		F	75	Yes	Yes	<1	Yes	Yes	No	Conscious	Yes	No
		M	23	No	No	<1	No	Yes	No	Conscious	No	No
		F	30	No	No	<1	No	Yes	No	Unconscious	No	No
		M	65	No	Yes	<1	No	Yes	No	Conscious	Hemiplagia	No
118	Boussarsar 2020 [146]	M	43	Yes	No	NA	No	Yes	No	Unconscious	No	No
		M	70	Yes	Yes	NA	No	Yes	No	Unconscious	No	No
		M	82	No	No	NA	No	Yes	No	Unconscious	No	No
119	Ameen 2011 [23]	M	16	No	No	<1	Yes	Yes	No	Unconscious	No	No
		M	49	No	Yes	<1	No	Yes	No	Unconscious	Yes	Yes
120	Howard 2016 [147]	M	77	No	No	NA	Yes	Yes	No	Unconscious	No	No
		M	60	No	No	NA	Yes	Yes	No	Unconscious	No	No
		F	49	No	No	NA	Yes	Yes	No	Unconscious	No	No
		F	55	No	No	NA	No	Yes	No	Unconscious	No	No
121	Thacker 2017 [148]	F	37	No	No	NA	Yes	Yes	No	Conscious	Riht hemiplagia	No
		F	45	No	No	NA	Yes	Yes	No	Conscious	Riht hemiplagia	No
		M	55	No	No	NA	No	Yes	No	Conscious	Riht hemiplagia	No
122	Fidalgo 2022 [149]	M	60	NA	NA	NA	No	Yes	No	NA	NA	NA
		M	68	NA	NA	NA	No	Yes	No	NA	NA	NA
		M	53	NA	NA	NA	No	Yes	No	NA	NA	NA
		M	71	NA	NA	NA	No	Yes	No	NA	NA	NA
		F	61	NA	NA	NA	Yes	No	No	NA	NA	NA
		F	66	NA	NA	NA	Yes	No	No	NA	NA	NA
		F	78	NA	NA	NA	Yes	No	No	NA	NA	NA
		F	80	NA	NA	NA	Yes	No	No	NA	NA	NA
123	Howard 2019 [150]	M	76	No	No	<1	Yes	Yes	No	Conscious	No	No
		M	77	No	No	<1	Yes	Yes	No	Unconscious	No	No
		F	58	No	No	<1	Yes	Yes	No	Conscious	Hemiparesis	Yes
		F	49	No	No	>1	Yes	Yes	No	Conscious	No	No
		F	30	No	No	>1	Yes	Yes	No	Conscious	No	No
		F	68	No	No	>1	Yes	Yes	No	Conscious	No	No
124	Yao 2023 [151]	M	59	Yes	Yes	<1	No	Yes	No	No	No	No
		F	63	Yes	Yes	<1	No	Yes	Yes	No	No	Yes
		M	83	Yes	No	<1	No	Yes	No	Unconscious	Left hemiparesis	No
125	Kanbayashi 2016 [22]	M	77	Yes	No	NA	Yes	Yes	No	Unconscious	Hemiparesis	Yes
		M	15	Yes	Yes	NA	No	No	No	Conscious	No	No
		M	45	No	No	NA	No	No	No	Conscious	No	No
		M	38	No	No	NA	Yes	No	No	Conscious	No	No
		F	61	No	No	NA	Yes	No	No	Conscious	No	No
		F	83	No	No	NA	Yes	No	No	Conscious	No	No
126	Karasu 2022 [152]	M	11	No	No	<1	Yes	Yes	Yes	Unconscious	No	No
		M	6	No	No	<1	No	Yes	Yes	Unconscious	No	No
127	Ogul 2022 [153]	M	55	Yes	Yes	NA	NA	NA	NA	NA	NA	NA
		M	62	Yes	No	NA	NA	NA	NA	NA	NA	NA
		M	77	Yes	No	NA	NA	NA	NA	NA	NA	NA
		M	68	Yes	No	NA	NA	NA	NA	NA	NA	NA
		M	80	Yes	No	NA	NA	NA	NA	NA	NA	NA
		M	58	Yes	No	NA	NA	NA	NA	NA	NA	NA
		M	69	Yes	No	NA	NA	NA	NA	NA	NA	NA
		F	72	Yes	No	NA	NA	NA	NA	NA	NA	NA
		F	81	No	No	NA	NA	NA	NA	NA	NA	NA
		F	54	No	No	NA	NA	NA	NA	NA	NA	NA
		F	66	No	No	NA	NA	NA	NA	NA	NA	NA
128	Osborn 2010 [13]	F	82	No	No	<1	Yes	Yes	No	Conscious	NA	No
		F	52	No	No	<1	Yes	Yes	No	Conscious	NA	No
		F	44	No	No	<1	Yes	Yes	No	Conscious	NA	No
		F	45	No	No	<1	No	Yes	No	Unconscious	NA	No
		F	70	No	No	<1	No	Yes	No	Conscious	NA	No
		F	49	No	No	<1	No	Yes	No	Conscious	NA	No
		F	72	No	No	<1	No	Yes	No	Conscious	NA	No
		F	63	Yes	No	<1	Yes	Yes	No	Unconscious	NA	No
		F	93	No	Yes	<1	Yes	Yes	No	Conscious	NA	No
		F	71	No	No	<1	No	Yes	No	Conscious	NA	Yes
		F	44	No	No	<1	No	Yes	No	Conscious	NA	No
		F	48	No	No	<1	No	Yes	No	Conscious	NA	No
		F	49	Yes	No	<1	Yes	Yes	No	Unconscious	NA	No
		F	62	Yes	No	<1	No	Yes	No	Conscious	NA	No
		F	31	Yes	Yes	<1	No	Yes	No	Conscious	NA	No
		M	47	Yes	No	<1	No	Yes	No	Conscious	NA	No
		M	88	Yes	No	<1	No	Yes	No	Conscious	NA	No
		M	77	No	Yes	<1	No	Yes	No	Conscious	NA	No
		M	61	No	No	<1	No	No	No	Conscious	NA	No
		M	65	No	No	<1	No	No	No	Conscious	NA	No
		M	50	No	No	<1	No	No	No	Conscious	NA	No
		M	34	No	No	<1	No	No	No	Conscious	NA	No
		M	41	Yes	No	<1	No	No	No	Conscious	NA	No
		M	59	No	No	<1	No	No	No	Conscious	NA	No
		M	77	Yes	No	<1	No	No	No	Conscious	NA	No
		M	82	Yes	No	<1	No	No	Yes	Conscious	NA	No
		M	28	No	No	<1	No	No	No	Conscious	NA	No
		M	72	No	No	<1	No	No	No	Conscious	NA	No
		M	62	No	No	<1	No	No	No	Conscious	NA	No
		M	66	No	No	<1	No	No	No	Conscious	NA	No
		M	93	No	No	<1	No	No	No	Conscious	NA	No
		M	71	No	No	<1	No	No	No	Conscious	NA	No
		NA	NA	No	No	<1	No	No	No	Conscious	NA	No
		NA	NA	No	No	<1	No	No	No	Conscious	NA	No
		NA	NA	No	No	<1	No	No	No	Conscious	NA	No
		NA	NA	No	No	<1	No	No	No	Conscious	NA	No
		NA	NA	No	No	<1	No	No	No	Conscious	NA	No
129	Legriel 2014 [154]	F	83	Yes	No	<1	No	Yes	No	Unconscious	Left hemiparesis	No
		F	67	No	No	<1	No	Yes	No	Unconscious	No	No
		F	72	Yes	No	<1	Yes	Yes	No	Unconscious	Left hemiparesis	No
130	Shah 2018 [21]	M	55	Yes	No	>1	Yes	Yes	No	Conscious	No	No
		M	67	No	No	>1	Yes	Yes	No	Conscious	No	No
		M	71	No	Yes	>1	Yes	Yes	No	Unconscious	Motor deficit	No
		M	71	Yes	No	>1	Yes	Yes	No	Conscious	Motor deficit	Yes
		M	81	Yes	Yes	>1	Yes	Yes	No	Conscious	No	Yes
		M	76	Yes	Yes	>1	Yes	Yes	No	Conscious	No	No
		M	62	No	No	>1	Yes	Yes	No	Conscious	No	No
		F	43	Yes	No	>1	Yes	Yes	No	Conscious	Motor deficit	No
		F	57	No	No	>1	Yes	No	No	Conscious	No	No
		F	89	No	No	>1	Yes	Yes	No	Conscious	No	No
		F	52	Yes	No	>1	Yes	Yes	No	Conscious	No	No
		F	52	Yes	No	>1	Yes	Yes	No	Conscious	Motor deficit	No

Following corresponding missing data exclusion, SPSS software version 26 was utilized for analyzing documented variables. The mean patient age was 59 years (Table 2), with 158 males constituting 57,7% of the total population. Among the population, 125 patients (46.8%) had hypertension, 56 (21.3%) had diabetes mellitus.

**Table 2:** Demographic characteristics of the patients included in the study.

Feature	Frequency
No. of Patients	Total = 279 (M = 158, F = 121)
Mean Age (Range)	59 years (1 month - 93 years)
Co-morbidities	Hypertension = 125/ 279 (46.8%) Diabetes Mellitus = 56/ 279 (21.3%)

Key: M = males, F = females.

Regarding symptoms, the mean duration of symptoms was shorter than a day. Visual disturbance was reported in 116 patients (43,9%), altered mental state in 207 (77,2%), and memory impairment in 15 (5,3%). Additionally, 39 patients (14,6%) experienced slurred speech, motor deficits and dyscoordination were found in 79 patients (35.6%).

Ischemia was the cause in 249 cases (96.1%), with minor arterial disease in 2 (0.8%), stenosis in 3 (1.2%), perforation in 1 (0.4%), and other etiologies in 4 (1.5%). The paramedian thalamus alone was involved in 100 patients (38,5%), the midbrain alone in 22(7.8%), both paramedian thalamus and midbrain in 86 patients (33.1%) and multiple sites in 52 (20%). Thalamic blood supply distribution was documented in some article and the prevalence was analyzed as: 81% type I, 9,5% type IIa, and 4.8% type IIb, 4,8% type III (Table 3).

**Table 3:** Clinical presentation, etiology, and the vascular supply to the thalamus.

Feature	Frequency
Symptoms	Visual Disturbance = 116 (43.9%)
	Altered Mental State = 207 (77.2%)
	Memory Deficit = 15 (5.3%)
Etiology	Ischemia = 249 (96.1%)
	Perforation = 1 (0.4%)
	Stenosis = 3 (1.2%)
	Minor Arterial Disease = 2 (0.8%)
	Others = 4 (1.5%)
Sites Involved	Paramedian thalamus alone:100 (38,5%)
	Midbrain alone in 22 (7.8%)
	Both paramedian thalamus and midbrain in 86 patients (33.1%)
	Multiple sites in 52 (20%)
Variant of Thalamic Blood Supply	Type I: 17 (81%)
	Type IIa: 2 (9,5%)
	Type IIb: 1 (4,8%)
	Type III: 1 (4,8%)

Treatment-wise, 129 (85,4%) were managed conservatively, 17 patients (11,3%) underwent thrombolytic therapy, 4 (2.6%) underwent mechanical thrombectomy, and 1 (0.7%) received a combination of thrombolysis and mechanical thrombectomy (Table 4). Recovery rates post-exclusion of missing data showed complete recovery in 51 patients (24.8%), mild deficits in 110 (53.4%), severe deficits in 35 (17%), and death in 10 (4,9%). The mean recovery time was 11,5 days, and follow-up duration averaged 13.7 months.

**Table 4:** Treatment options and follow-up.

Feature	Frequency of single treatment modality
Therapy	Conservative = 129 (85,4%)
	Thrombolytic = 17 (11,3%)
	Mechanical Thrombectomy = 4 (2.6%)
	Combined* = 1 (0.7%)
Duration of Follow-up	1 to 55 months

Key: *Combination of thrombolysis and mechanical thrombectomy.

**Fig. 2:**
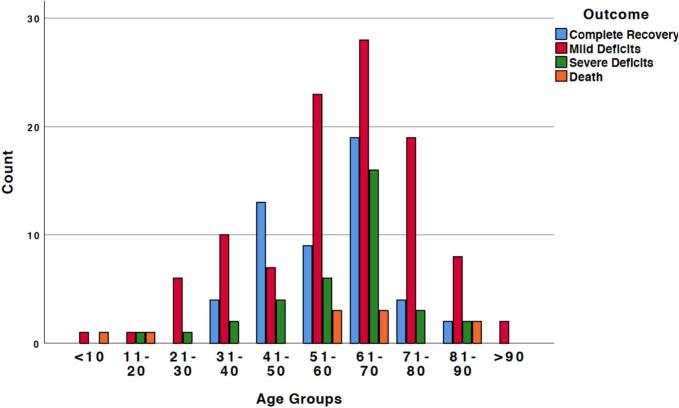
showing the outcomes according to the age groups.

Statistical significance via the Pearson Chi-Square test with a (*p*-value<0,05) indicated several associations: (A) Patients with arterial hypertension and other cardiovascular diseases were inclined to develop severe deficits. (B) Higher risk of developing deficits was found with involvement of the paramedian thalamus more than the midbrain, which tremendously increases with involving both of them. (C) Patients presenting with anisocoria tend to have more deficits at the last follow up than those presented without it. (D) The best age group to recover completely is those between 41 and 50 years old, they have more complete recovery rat than any other group while the worst (Fig. 2). (E) altered consciousness at the time of administration tends to develop more severe symptoms.

## 4 Discussion

The classic categorization of the thalamic vascular supply delineated into four distinct territories—namely, anterior, paramedian, inferolateral, and posterior—showcases the complex and overlapping nature of this intricate vascular network within the brain. Gérard Percheron's pivotal 1973 discovery of the AOP introduced a fascinating anomaly within this established framework. Originating from segment one (P1) of the posterior cerebral artery (PCA) and observed in a minority of individuals (approximately 4%–12% of the population). The AOP uniquely supplies both sides of the paramedian thalamus and the upper midbrain through a single shared branch. This underscores the potential interplay and connectivity between the traditionally categorized thalamic territories, highlighting the significance of such rare anatomical variations in understanding the broader thalamic vascular supply [3,7].

**Fig. 3:**
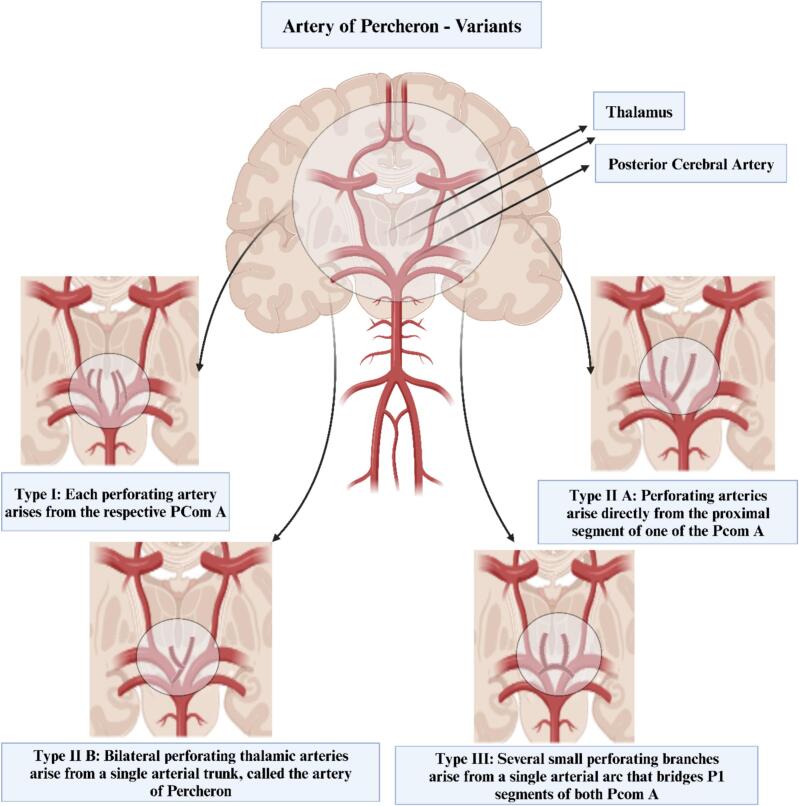
Variants of the artery of Percheron.

Percheron identified four types of paramedian perforating arteries to the thalami (Fig. 3). The first variant (type I), involves the arteries emerging from the proximal segments of both PCAs on each side. The second type (type IIa), occurs when the arteries arise directly from the proximal segment of just one PCA. However, in some people, a single arterial trunk stems off the P1 segment of one of the PCAs and this trunk then divides to supply both thalami and the upper midbrain (type IIb); this is the AOP. Lastly, Type III is defined by the presence of a single arterial arc that links the proximal segments of both PCAs and from this arc, the paramedian thalamic perforating arteries arise [[7], [8], [9]]. In this study, we elucidated that the type I variant was most prevalent among 81% of the population, while type IIa was observed in only 9.5%. The rarest variant type IIb and type III, were found in just 4.8% of the individuals examined.

**Fig. 4:**
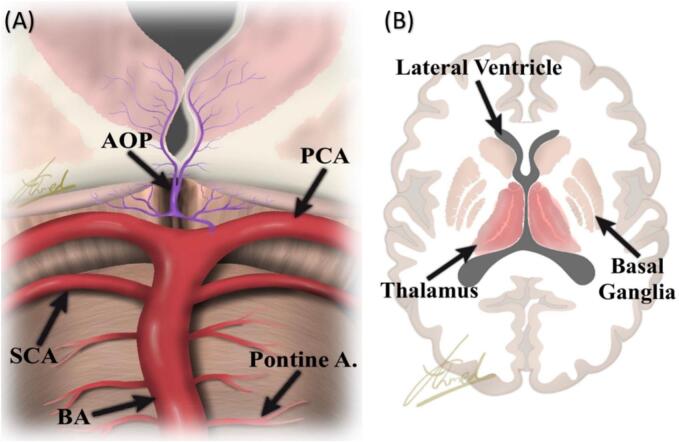
Illustration of AOP with infarction from anatomical perspective.

Artery of Percheron occlusion results in a Percheron infarction, marked by specific bilateral paramedian thalamic distribution potentially alongside a mesencephalic distribution [10] (Fig. 4). With a relatively small ischemic lesion in the bilateral paramedian thalami, patients with Percheron infarction would present with an apparent life-threatening event comprising a massive ischemic infarction unless prompt intervention is administered [11,12]. Although likely underestimated, the prevalence of AOP is only 0.1% to 2% and 4% to 18% of all and thalamic strokes, respectively [3,13]. Similarly, Bogousslavsky analyzed 1000 consecutive patients sustaining their first episode of stroke and found that isolated thalamic infarcts, as a presenting feature, comprised 11% of all strokes in the posterior circulation while midbrain ischemic infarctions constituted 7% only [14]. In clinical practice and imaging, Percheron infarction must therefore be considered for its management. A few isolated cases have been reported in clinical practice in the previous decades [12,15]. N.A. Lazzaro et al. and Antonio Arauz et al. successively demonstrated the clinical and imaging aspects of Percheron infarction in 37 and 15 cases, respectively [3,13].

Percheron infarction is a catastrophic cerebral vascular due to its impact on the blood supply to the paramedian thalamus and midbrain. Zhihua Xu et al., found that for patients with acute ischemic infarction, the occurrence of Percheron infarction was 0.27% [16]. Furthermore, researchers have demonstrated that 0.1%–0.4% of all patients with first episode of acute ischemic stroke sustained a Percheron infarction [3,17,18]. Our findings correlate with prior studies in the literature. Percheron infarction, although rare, usually presents with an apparent life-threatening event. However, the initial and subsequent symptoms are variable. Therefore, it is difficult for a neurologist to diagnose this condition in a timely manner with clinical observations alone. Moreover, there is no predilection to sex, race-ethnicity, and age in the reported cases of AOP stroke in the literature [18,19]. Our study findings, aligned with those of Garcia-Grimshaw et al., indicate that the characteristics of AOP stroke, including age distribution and gender predominance, may vary based on its etiology [19]. Much like Lin (P.C.), our research highlights a wide occurrence of AOP stroke across various age groups, with a notable concentration observed within the 30 to 70-year range [20]. Similar to our study, Stamm (B.J.) et al. and Suzuki (K.)et al. found a slight male predominance in patients with AOP stroke, aligning with the general trend observed in prior research [21,22].

In our study, the risk factors for Percheron infarction included hypertension, hyperhomocysteinemia, recent history of smoking, Diabetes Milletus (DM), and hyperlipidemia [3,23]. Specifically, among the patients examined, a substantial portion of the patients (46.5%) had pre-existing hypertension, with the majority being males (57,7%). Furthermore, DM was reported as a risk factor in 56 (21.3%) out of 280 patients.

Expanding on the risk factors, Saez et al. highlighted distinct patterns in thalamic strokes across different age groups. They found that in younger patients, cigarette smoking was the primary risk factor. In contrast, for individuals aged 45 and above, hypertension emerged as the predominant risk factor, attributed to its association with atherosclerosis. This observation signifies a shift in prominent risk factors with age, evident across various stroke types, including Percheron infarction [24].

Our study on Percheron infarction's etiology resonates with diverse perspectives from Arauz et al. and de la Cruz-Cosme et al. Arauz et al. identified small vessel disease as the primary cause [3], while de la Cruz-Cosme et al. emphasized a dual association with cardio-embolism and cardiovascular diseases (CVD), including conditions like stenosis [25]. These differences might influence the response to variations in region or race. Generally, the most common etiologic factors for Percheron infarction are small vessel disease and cardio-embolism [3,25].

Analyzing our findings, 96.1% of the cases were attributed to ischemia, indicating the multifaceted origins of this condition. Contributing factors encompassed perforation (0.4%), stenosis (1.2%), minor arterial disease (0.8%), CVD (including both cardio-embolism and other cardiac-related etiologies) and other factors (1.4%). These factors support the prevalent recognition of small vessel disease, CVD, and ischemia as primary etiological factors in Percheron infarction, highlighting the intricate pathways leading to its occurrence.

Lazzaro et al. identified four distinct ischemic infarction patterns arising from AOP occlusion based on their review of 37 patients, by evaluating clinical presentations and imaging findings. Among their observations, the most common pattern (43%) demonstrated damage to both the paramedian thalami and midbrain, while 38% of the patients exhibited isolated paramedian thalamic injury. In approximately 14% of the cases, damage extended to the anterior thalamic nuclei in addition to the paramedian thalami and upper midbrain. A rarer pattern (5%) depicted bilateral paramedian and anterior thalamic damage without midbrain involvement [13]. In our study, the primary site of infarctions was aligned with Lazzaro et al. findings [13], with the paramedian thalamus being the most affected (38,5%), followed by both midbrain and paramedian thalamus involvement (46.9%). Other injury sites were less prevalent, accounting for only 20%**.**

Patients experiencing Percheron infarction encounter diverse initial symptoms. Some individuals exhibit unremarkable and atypical symptoms, like dizziness. On one hand, these subtle symptoms might be overlooked by certain physicians; on the other hand, patients might not prioritize seeking adequate care. Consequently, these circumstances may extend the duration between symptom onset and seeking medical attention. Additionally, accurately determining the precise timing of the ischemic infarction poses a challenge when establishing the thrombolytic therapy window. Furthermore, subsequent symptoms manifest differently in each case. Hence, relying solely on clinical observations renders timely diagnosis impossible, highlighting the necessity for early recognition and immediate imaging for this condition [16].

When suspecting Percheron infarction, prioritizing magnetic resonance imaging-diffusion-weighted imaging (MRI-DWI) sequences becomes crucial due to the clinical relevance of apparent diffusion coefficient (ADC) maps and DWI in timing these infarctions [26,27]. Zhihua Xu et al. conducted a study, in 18 cases, that demonstrated a 100% positivity rate for detection and localization of Percheron infarction using MRI-DWI sequence, while computed tomography (CT) exhibited negative results in 50% of the patients [16]. This underscores the pivotal role of the DWI-MRI sequence in accurately diagnosing Percheron infarction. Nonetheless, we emphasize the importance of conducting a CT scan upon admission to exclude brain hemorrhage [26,27]. Instances reported by Cassourret G et al. and Mecbure Nalbantoglu et al. noted an AOP occlusion in normal initial brain MRIs, including DWI sequences [28,29]. Therefore, considering a second MRI may be warranted if a strong clinical suspicion of Percheron infarction persists [16].

Until now, the diagnosis of Percheron infarction depended on lesions in a specific bilateral paramedian thalamic distribution with or without a mesencephalic distribution based on brain imaging. Other etiologies, such as top basilar syndrome, deep cerebral vein thrombosis, Wernicke's encephalopathy, and glioma, should be considered [16]. Eva Guy Rodriguez et al. indicated that patient history, specific imaging characteristics, and the presence or absence of lesions outside the thalami aid in narrowing the differential diagnosis [30].

Percheron infarction results from occlusion within the AOP, which is usually not visible on standard magnetic resonance angiography (MRA) scans. Zhiua conducted a study that did not demonstrate the typical AOP image using MRA, but instead found that patients with Percheron infarction lacked the posterior communicating artery (PCoA) on several scans. This absence might suggest a lack of the primary collateralization [16]. However, it could also indicate natural variations in the circle of Willis. These variations could exacerbate symptoms when combined with internal carotid artery stenosis [31]. Some evidence also suggested that PcoA hypoplasia may contribute to a propensity for thalamic lacunar stroke due to its dominant role in providing collateral supply to the proximal PCA territory [32]. However, the presence of anatomic variations like the AOP and PcoA hypoplasia? Could potentially lead to a hemodynamic infarction due to inadequate regional collateral blood flow [16].

The clinical manifestations of AOP stroke exhibit significant variability, encompassing various symptoms like bilateral vertical gaze palsy (65%), memory impairment (58%), and coma (42%) [19,33], as well as other reported features such as hypersomnolence (29%), akinetic mutism and behavioral disorders like apathy, agitation, and aggressiveness [18,19,33]. When midbrain involvement occurs, the clinical presentation often includes hemiplegia, movement disorders, cerebellar ataxia, and oculomotor disturbances, in conjunction with the aforementioned triad [16,18].

Bithalamic stroke is closely linked to the thalamus, pivotal in sleep regulation and arousal maintenance. Interruption of noradrenergic and dopaminergic impulses from the ascending reticular activating system to the thalamus contributes to hypersomnolence post-stroke [34,35]. Bilateral thalamic infarcts result in more pronounced sleep-wake disturbances compared to unilateral infarcts [36], often leading to increased sleep needs [35,36].

In this study, 43.9% of our patients presented with visual disturbance and gaze palsy, which resonates with the common bilateral vertical gaze palsy described in previous studies [[34], [35], [36]]. Moreover, altered mental status was prevalent in 77.2% of our cases, a percentage consistent with the documented high incidence of coma in AOP strokes. However, memory deficits were observed in 5.3% of our cases, which is lower compared to the literature [19,33]. Furthermore, less frequent symptoms in our study motor deficits and dyscoordination were found in 79 patients (35.6%) and slurred speech (14.6%).

Understanding the relationship between imaging findings and the diverse clinical presentations of Percheron infarction could significantly improve its recognition and subsequent management. This analysis would play a crucial role in guiding both the diagnosis and the selection of appropriate treatment strategies for this condition, enhancing our comprehension of its complexities and potential complications.

Among cases managed for acute AOP infarction, our findings reveal notable associations with different treatment modalities. Thrombolytic therapy, specifically within a time window of <4.5 to 6 h, demonstrated a substantial link to achieving complete recovery and mild residual deficits during the final follow-up, affirming its status as the most effective treatment for acute AOP infarction [37]. This therapy aims to promote recanalization, aligning with the current goal of managing acute AOP occlusion [38]. Conversely, conservative management, observed in 85.4% of cases, also showed a significant portion achieving complete recovery [39]. A minimal percentage (2.6%) of the patients in our study underwent mechanical thrombectomy, which exhibited a significant correlation with mild residual deficits observed during the last follow-up. The prominence of thrombolytic therapy within the critical time window underscores its efficacy and highlights the importance of prompt intervention in treating infarctions involving the AOP [39].

## 5 Conclusion

In conclusion, our study sheds light on acute artery AOP infarction, a rare but clinically significant cerebrovascular condition. Through meticulous analysis of patient-level data from 130 papers involving 279 individuals, we have identified common clinical characteristics, etiological factors, and treatment outcomes. Our findings reveal a male predominance, with hypertension and diabetes mellitus being common comorbidities among the studied population. Symptomatically, visual disturbance, altered mental state, and motor deficits were prevalent presentations, with ischemia, particularly involving the paramedian thalamus, identified as the primary cause. Furthermore, we uncovered significant associations between various factors and the development of severe deficits, emphasizing the importance of early recognition for prompt management. Thrombolytic therapy within the critical time window emerged as the most effective treatment modality, showing significant associations with favorable outcomes. Overall, our study contributes to a deeper understanding of AOP infarction and provides insights for improved diagnosis and treatment strategies in clinical practice. Further research is warranted to validate these findings and explore additional aspects of this condition.

## CRediT authorship contribution statement

**Oday Atallah:** Writing – review & editing, Writing – original draft, Supervision, Methodology, Conceptualization. **Yasser F. Almealawy:** Writing – review & editing, Writing – original draft. **Arwa Salam Alabide:** Writing – original draft, Formal analysis. **Minaam Farooq:** Writing – review & editing, Writing – original draft. **Vivek Sanker:** Writing – review & editing, Writing – original draft. **Suraa N. Alrubaye:** Writing – review & editing, Writing – original draft. **Rami Darwazeh:** Writing – review & editing, Writing – original draft. **Wireko Andrew Awuah:** Writing – review & editing, Writing – original draft. **Toufik Abdul-Rahman:** Writing – review & editing, Writing – original draft. **Ahmed Muthana:** Writing – review & editing, Writing – original draft. **Aalaa Saleh:** Writing – review & editing, Writing – original draft. **Jack Wellington:** Writing – original draft, Supervision. **Amr Badary:** Writing – review & editing, Writing – original draft, Methodology, Formal analysis.

## Funding

This research was conducted without external funding or grants

## Declaration of competing interest

None. (This research was conducted without external funding or grants).
